# Role of environmental enrichment on social interaction, anxiety, locomotion, and memory in Wistar rats under chronic methylphenidate intake

**DOI:** 10.3389/fnbeh.2023.1251144

**Published:** 2023-11-14

**Authors:** Laura Herrera-Isaza, Santiago Zárate-Guerrero, Karen Corredor, Ángela Gómez-Fonseca, Guillermo Escobar-Cornejo, Fernando P. Cardenas

**Affiliations:** Laboratory of Neuroscience and Behavior, Department of Psychology, Universidad de los Andes, Bogotá, Colombia

**Keywords:** methylphenidate, psychostimulants, social interaction, anxiety, enriched environment, ADHD, rat

## Abstract

**Introduction:**

Chronic use of various compounds can have long-lasting effects on animal behavior, and some of these effects can be influenced by the environment. Many environmental enrichment protocols have the potential to induce behavioral changes.

**Aim:**

The aim of the present study was to investigate how environmental enrichment can mitigate the effects of chronic methylphenidate consumption on the behavior of Wistar rats.

**Methods:**

The animals were housed for 20 days under either an environmental enrichment protocol (which included tubes of different shapes) or standard housing conditions. After seven days, half of the rats received 13 days of oral administration of methylphenidate (2 mg/kg). After seven days, the rats underwent behavioral tests, including the elevated plus maze (anxiety), open field (locomotion), object-in-place recognition test (spatial memory), and a test for social interaction (social behavior).

**Results:**

The results showed that the enriched environmental condition reversed the enhanced time in the open arms of the elevated plus maze induced by methylphenidate (F_[1,43]_ = 4.275, *p* = 0.045). Methylphenidate also enhanced exploratory rearing in the open field (F_[1,43]_ = 4.663, *p* = 0.036) and the time spent in the open area of the open field (H[3] = 8.786, *p* = 0.032). The enriched environment mitigated the inhibition of social interaction with peers induced by methylphenidate (H[3] = 16.755, *p*  < 0.001) as well as the preference for single exploratory behavior (H[3] = 9.041, *p* = 0.029).

**Discussion:**

These findings suggest that environmental enrichment can counteract some of the effects of methylphenidate. These results are relevant for the clinical treatment of the long-lasting secondary effects associated with methylphenidate pharmacological treatment.

## Introduction

“Methylphenidate (MPH) is the most commonly prescribed pharmacological treatment for attention deficit hyperactivity disorder (ADHD) and belongs to a class of psychostimulant drugs (Jaeschke et al., 2021). Psychostimulant drugs have the potential to impact behavior, cognition, and emotional functions in both animals and humans (Anghelescu and Ahlers, 2020). When administered to humans, psychostimulants can lead to various behavioral effects, including euphoria and alterations in cognitive or physical performance; the specific effects depend on several factors such as the type of psychostimulant (e.g., cocaine, amphetamine, and methylphenidate), route of administration, and the individual’s condition at the time of drug intake (Koob et al., 2020). While MPH can significantly improve the functioning of brain structures affected by ADHD, it may have detrimental effects on other areas (Coon et al., 2014). Furthermore, under certain conditions, the use of MPH can increase the risk of substance abuse (Linssen et al., 2014). These considerations highlight the need to investigate potential adverse effects associated with unnecessary consumption of psychostimulant drugs, including MPH as the most prescribed medication for ADHD.

Additionally, there is a growing interest in examining other factors that may mitigate the consequences of chronic MPH use, such as environmental enrichment, which is recognized as a protective factor against substance abuse (Rodríguez-Ortega and Cubero, 2018) and as a useful tool in reversing the emotional and behavioral effects induced by early life stressful situations (Corredor et al., 2022).

It has been demonstrated that low doses of psychostimulants such as MPH can improve attention and memory processes in both humans and rats (Spencer et al., 2015). According to the same authors, low doses are the ones used clinically to treat attention deficits in humans. For the pharmacological treatment of ADHD in humans, a low dose of MPH is effective in improving attention and memory by addressing prefrontal cortex hypoactivity (Cheng et al., 2014). However, high doses of the same drug have been shown to lead to aggressive behaviors and other behavioral and cognitive impairments (Smith and Farah, 2011). Environmental enrichment has also demonstrated the ability to mitigate the effects of stress on memory and cognitive functions, thereby enhancing their development (Macartney et al., 2022).

MPH consumption can not only affect cognitive processes but also interfere with emotional processing, such as anxiety. It has been reported that MPH can reduce anxiety levels, unlike other stimulants such as methamphetamines (Boyette-Davis et al., 2018). This potential anxiolytic effect is supported by reports on the enhanced time spent in the open arms of the elevated plus maze after MPH administration (Martin et al., 2018). However, it is important to consider that the anxiolytic effect of methylphenidate can vary depending on the baseline anxiety levels of each animal prior to consumption (Segev et al., 2016). In contrast, there is also evidence that in some cases, methylphenidate consumption can increase anxiety in animals, and this effect can be mitigated by physical activity or forced exercise in the animals (Motaghinejad et al., 2015). The evidence regarding the consumption of psychostimulants and their impact on anxiety is extensive and, at times, contradictory. Therefore, it is important to continue studying this topic. There is an interest in investigating whether other environmental variables, such as environmental enrichment, can mitigate the effect of chronic MPH consumption on anxiety.

Social interaction is another area of significant behavioral impact of psychostimulants. The social environment of an animal, including humans, directly influences its development (Grigoryan et al., 2022). Consequently, in recent years, the social behavior of animals has been extensively studied (Hackenberg et al., 2021), with many of these studies being conducted on rats. In the social behavior of rats, the mere proximity between individuals is a good predictor of the intention to interact. It is known that social behavior can be inhibited by MPH consumption (Vanderschuren et al., 2008). Conversely, other variables, such as environmental enrichment, have shown to promote the development of social behavior in rats by facilitating social responses between individuals (Neal et al., 2018). Consequently, there is an increasing interest in studying the effect of environmental enrichment on human social behavior and its therapeutic potential in treating neurodevelopmental problems (Ball et al., 2019; Corredor et al., 2022).

Furthermore, several studies have demonstrated that psychostimulants, while suppressing social behavior in animals, can also modify locomotion and increase rearing behavior (Šlamberová et al., 2015). Specifically, it has been shown that MPH consumption can increase locomotion in animals both in open-field tests and in their normal home-cage activity (Martin et al., 2018). These changes in locomotion can be explained by observed alterations in the animals’ dopaminergic system resulting from chronic methylphenidate treatment and the environmental conditions (standard vs. enriched) in which they are raised (Gill et al., 2013). Environmental enrichment has been shown to influence animals’ sensitization to modify their locomotion in response to methylphenidate (Malone et al., 2022). Given the potential of this variable, it is relevant to continue studying whether an enriched environment can reverse the effects of a psychostimulant on animal locomotion.

The present study aims to analyze the role of environmental enrichment on chronic, low-dose consumption of the psychostimulant methylphenidate in relation to anxiety, memory, social interaction, and locomotion in Wistar rats. To achieve this, animals receiving chronic treatment with MPH were housed in either a standard or enriched environment, and behavioral tests for anxiety, locomotion, exploration, and social interaction were conducted.

## Method

### Animals

A total of forty-seven male Wistar rats were utilized for this experiment. The rats were derived from a Wistar strain from Charles River Laboratories and were grouped in sets of four individuals and placed in polycarbonate cages (16.5 cm × 50 cm × 35 cm). The cages were maintained in a controlled environment featuring a 12-h light/12-h dark cycle (lights on at 06:00), ad libitum availability of food (Zeigler 104) and water, a consistent room temperature of 22 ± 2°C, and humidity maintained at 57 ± 10%. Due to the fact that the interest of this work is not the comparison of data between males and females, females were not included in the experiments in order to avoid possible hormonal interferences in the effects of methylphenidate and exposure to enriched environments. In fact, there are reports that show complex effects of the enriched environment on females, possibly due to hormonal factors (Corredor et al., 2022). Random assignment was conducted to allocate the rats into two different housing conditions: standard housing conditions (St) and an enriched environment (Ee). Each housing condition group was further divided into two subgroups: one receiving methylphenidate administration (MPH) and the other receiving vehicle administration (Vh). The rats were housed in their respective conditions from postnatal day 25 (PND25) to PND46. Throughout the study, the rats had unrestricted access to food and water.

For the social interaction experiments, an additional twenty-four animals from the same facility were utilized as “external peers.”

All experimental procedures were carried out in compliance with the Guide for the Care and Use of Laboratory Animals and were approved by the Institutional Animal Care and Use Committee of the Universidad de Los Andes.

### Drugs

Methylphenidate (Ritalin) was administered orally in a dose of 2 mg/Kg in syrup presentation. The animals in the control groups received only vehicle. To administer the drug to the animals, the tablet was crushed daily, and a 2 mg/kg dose was measured and diluted in 0.05 mL of a water and honey syrup. Subsequently, each rat’s weight was recorded, and the dose was calculated accordingly. Once the required amount was determined, it was drawn into a syringe and orally administered to each rat. This method proved effortless, as the rats readily consumed the entire dosage. The animals that were part of the group that did not receive the drug also received the honey and water solution using the same procedure. Several studies that have allowed a washout period of 3 to 15 days from the last dose of Ritalin have demonstrated that the behavioral effects of the drug persist beyond this period (Askenasy et al., 2007).

### Procedure

At postnatal day 25 (PND25), the subjects were placed in their designated housing condition. The enriched condition consisted of circular platforms and squared or circular PVC pipe parts, which were alternated every other day to prevent habituation, following the environmental enrichment protocols established in the Laboratory of Neuroscience and Behavior of the University of Los Andes. This involves using three distinct PVC pipe shapes to modify cage structures, offering novel surfaces and hiding spots for interaction with new items. To prevent habituation, objects are rotated every 3 days within the housing box. This form of enrichment is considered passive, as it does not require direct animal interaction. The standard condition animals were housed without any additional elements inside their cages.

After a period of 7 days (PND32), the animals were randomly assigned to one of the two treatment groups: methylphenidate or vehicle. The administration of methylphenidate or the vehicle began at this point and continued once a day for a duration of 13 days, until PND45. The drug was administered orally in syrup form, chosen for its pleasant taste and to minimize any stress associated with alternative administration methods. Following the completion of the drug administration, a six-day withdrawal period was provided to the animals to avoid immediate withdrawal effects.

**Figure 1:**
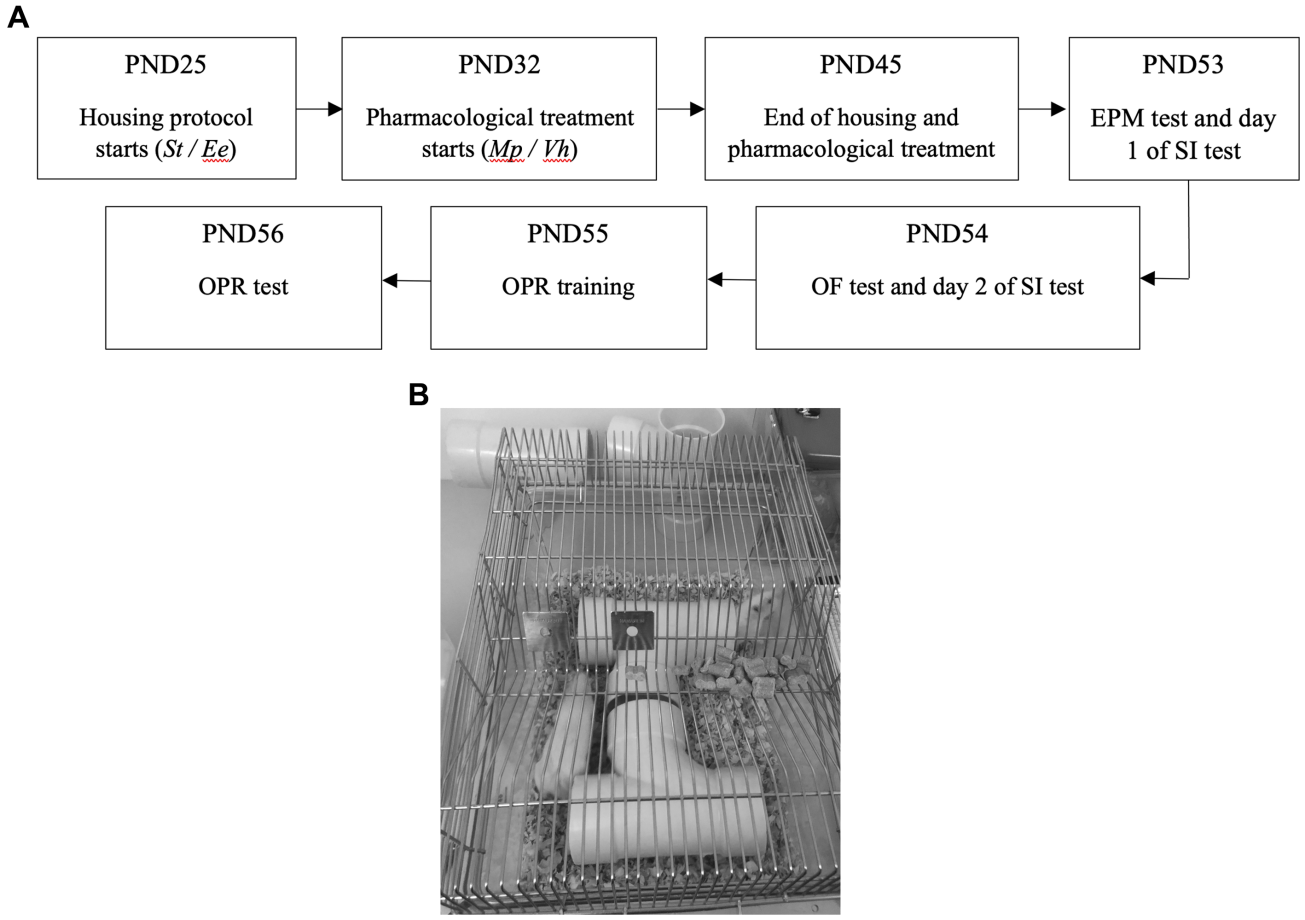
**(A)** Outline of the experimental timeline. PND: Post-natal day; St: Standard; Ee: Enriched environment; Mp: Pethylphenidate; Vh: Vehicle; EPM: Elevated plus maze; SI: Social interaction; OF; Open field; OPR: Object in place recognition; **(B)** Typical enriched environment.

Subsequently, all animals underwent behavioral tests as outlined in Figure 1, which describes the procedure.

### Behavioral testing

#### Elevated plus maze

On postnatal day 53 (PND53), all the animals underwent the Elevated Plus Maze (EPM) test. The maze comprises two open platforms intersected at right angles by two enclosed platforms with 40 cm high walls, forming open and closed arms. Each arm measures 50 cm in length and 10 cm in width. A 1 cm high plexiglass rim prevents rat falls. Constructed from wood coated with black melamine, the maze is elevated 50 cm above the floor, positioned in a secluded test room illuminated by white LEDs (60 Lux at the center). Rats, unexposed to the maze previously, were individually tested, starting in the center, and exploring for 5 min. After testing, rats returned to their cages, and the maze was cleaned using 10% alcohol and disposable towels. Digital recordings of the experiment were analyzed, tracking both arm entries and time spent. An arm entry was defined as placing all four paws on the arm’s surface. Each subject was placed in the center of the maze and allowed to freely explore for a duration of 5 minutes. The time spent and the number of entries into both the open and closed arms were recorded. Additionally, other behaviors such as grooming, rearing, head dipping, and stretching were also observed and recorded during the test.

#### Social interaction

Immediately after the Elevated Plus Maze (EPM) test, the animals entered the social interaction test. This test evaluates social interaction with an unfamiliar animal in a round open field and is used to assess social skills. Even low doses of psychostimulants have been reported to affect certain social behaviors (Meaney et al., 1981). Each experimental animal was placed in a cage with an unknown animal (external peer) and allowed to freely interact for 10 minutes. During this period, the frequency and latency of various social behaviors were recorded. These behaviors include: (1) Pinning, where one animal lies on its back with another animal on top; (2) Pouncing, which involves sniffing or rubbing the nose against the neck of another animal; (3) Contact, which includes grooming between peers and other forms of social exploration (Achterberg et al., 2015); (4) Exploration, representing moments of individual exploration; (5) Passing, when one animal passes over the other; and (6) Following, where one animal chases the other. These behaviors were selected to be observed with the intention of assessing the “natural” sociability of the animals, which is understood as non-violent interactions driven by the interest animals show in engaging with their peers (Vanderschuren et al., 2016). Some of these behaviors, including playful actions that are a part of the animals’ natural sociability and may be influenced by the environmental conditions in which the animals are situated, as well as other non-playful behaviors driven solely by an interest in interacting (Achterberg et al., 2014), were chosen for observation. After the social interaction test, the animals were returned to their home cages. The test was repeated 24-h later.

#### Open field

After a 24-h interval, the open field test was conducted. The animals were placed in a square plexiglass arena measuring 80 × 80 × 50 cm. Each subject was gently placed in the center of the open field and allowed to freely explore for a duration of 5 minutes. During this time, the amount of time spent in the center of the arena versus near the periphery, as well as the total distance covered, were recorded. After the completion of the five-minute exploration period, the animals were returned to their respective home cages.

#### Object in place recognition

Twenty-four hours after the open field test, the object in place recognition test was conducted to assess memory. This test is commonly used to evaluate learning and memory abilities.

The habituation phase took place on the first day, during which the animal was introduced to an open field measuring 80 × 80 × 50 cm and allowed to freely explore for 5 minutes. The purpose of this phase was to familiarize the animal with the test environment.

The following day, the animal was placed back into the same arena, this time with two identical objects positioned in specific locations near the center of the field. The animal was given time to explore the objects and their surrounding areas.

After a 24-h interval, the training phase commenced. This phase consisted of five trials, with the two identical objects being placed in the same positions throughout all trials. Visual cues were present in the field to provide spatial references.

24-h later, the testing phase began. During this phase, the location of one of the objects was changed while the other remained in its original position. The behavior of the animal, specifically the time spent circling the new location versus the old one, was recorded. It is important to note that the objects used in all tests were identical to ensure that the animal’s preference was based solely on the location rather than any physical characteristics of the objects.

The duration of the exploration behavior (direction of the animal’s snout and/or contact of the forepaws with the object) for each object was calculated. The following calculation was used:

$$\begin{array}{l} EI=\mathrm{Exploration}\;\mathrm{novel}\;\mathrm{object}\;\left(O1\right)-\mathrm{Exploration}\;\mathrm{familiar}\;\mathrm{object}\;\left(O2\right)/\\ \;\mathrm{Total}\;\mathrm{exploration}\;\mathrm{time}\;\mathrm{in}\;\mathrm{the}\;\mathrm{trial} \end{array}$$

## Data analysis

The collected data were analyzed using a two-way analysis of variance (ANOVA). Group mean differences were compared using the Student Newman–Keuls *post hoc* test, whenever necessary. If the data did not pass the normality test (Shapiro–Wilk) or exhibited unequal variances, non-parametric analysis (Kruskal-Wallis Analysis of Variance on Ranks) was conducted. In all instances, the alpha level was set at 0.05.

## Results

In Tables 1, 2, a summary of the statistical findings for the two-way ANOVA and the Kruskal-Wallis test (ANOVA on ranks) is provided when normality and variance tests failed.

**Table 1:** Statistical results of parametric analysis (Two Way ANOVA) for the performance in all the behavioral tests.

Behavior	Normality test (Shapiro–Wilk)	Equal variance test	Treatment (tt) (Vh / Mp)		Environment (hsg) (St / EE)		Interaction (tt × hsg)	
			F_[1,43]_	*p*	F_[1,43]_	*p*	F_[1,43]_	*p*
**Elevated plus-maze**								
Entries into the closed arms	0.586	0.533	6.545	0.014*	2.975	0.092	0.345	0.560
Time spent in closed arms	0.050	0.796	2.780	0.103	0.131	0.720	2.453	0.125
Entries into the open arms	0.491	0.707	2.840	0.099	1.577	0.216	3.269	0.078
Time spent in open arms	0.055	0.779	3.398	0.072	0.000	0.993	4.275	0.045*
Percentage of entries into the open arms	0.743	0.512	0.061	0.806	3.280	0.077	3.252	0.078
Crossings by the central square	0.149	0.873	6.830	0.012*	0.470	0.496	0.929	0.341
Time spent in the central square	0.477	0.390	0.246	0.622	0.719	0.401	0.030	0.864
Total distance run in the maze	0.087	0.629	6.187	0.017*	10.06	0.003*	0.239	0.627
Average speed while exploring the maze	0.084	0.619	6.467	0.015*	1.044	0.002*	0.250	0.619
**Object recognition test**								
Time spent exploring the new object	0.930	0.675	0.018	0.895	5.498	0.024*	0.186	0.886
**Social interaction test**								
Time of interaction with peers	0.625	0.066	0.213	0.647	0.713	0.403	0.309	0.581

Alpha value set in 0.05. Please refer to the text for *post hoc* comparisons. Vh, vehicle; Mp, methylphenidate; St, standard housing; EE, enriched environment; tt, treatment; hsg, housing. *Significant statistical difference.

**Table 2:** Statistical results of non-parametric analysis (Kruskal-Wallis ANOVA on ranks) for the performance in all the behavioral tests.

Behavior	Normality test (Shapiro–Wilk)	Equal variance test	H[3]	*p*
**Elevated plus maze**				
Total time of inactivity (different than freezing)	<0.050	<0.050	16.451	<0.001*
**Open field**				
Total time exploring the central area	<0.050	0.718	8.786	0.032*
Average of speed while exploring the periphery	<0.050	0.163	13.714	0.003*
Average of speed while exploring the central area	<0.050	0.194	2.302	0.512
Time spent self-grooming	<0.050	0.348	4.079	0.253
Time spent rearing	0.094	<0.050	9.625	0.022*
**Object recognition test**				
Time spent exploring the familiar object	<0.050	0.987	5.217	0.157
Index of exploration of the new object (time exploring new object / time exploring familiar object)	<0.050	0.480	6.146	0.105
**Social interaction test**				
Time pouncing	<0.050	<0.050	0.456	0.928
Rejection by pressing peers time	<0.050	<0.050	0.850	0.837
Time spent following peers (playing)	<0.050	<0.050	16.755	<0.001*
Time spent in single exploration	<0.050	0.641	9.041	0.029*

Alpha value set in 0.05. Please refer to the text for *post hoc* comparisons. *Significant statistical difference.

### Emotional response

**Figure 2:**
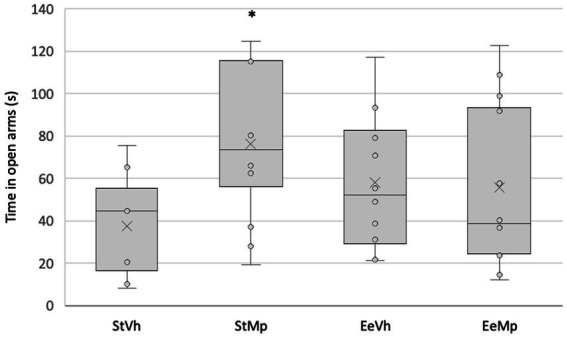
Time spent in the open arms of the elevated plus maze for all groups. Median represented by the inner line in the box and Average represented by the inner “x”. *: different from the pharmacological control in the same housing condition.

Regarding the time spent in the open arms of the elevated plus maze (see Figure 2), the ANOVA revealed significant differences (F_[1,43]_ = 4.275, *p* = 0.045) in the interaction between the factors (treatment × housing condition). *Post hoc* analysis of group means differences using the Student Newman–Keuls test showed that, for animals housed in the standard condition, those treated with methylphenidate spent a significantly longer time in the open arms compared to those receiving the vehicle. However, this effect was not observed in animals housed in the enriched environment condition.

### Exploration and locomotion

**Figure 3:**
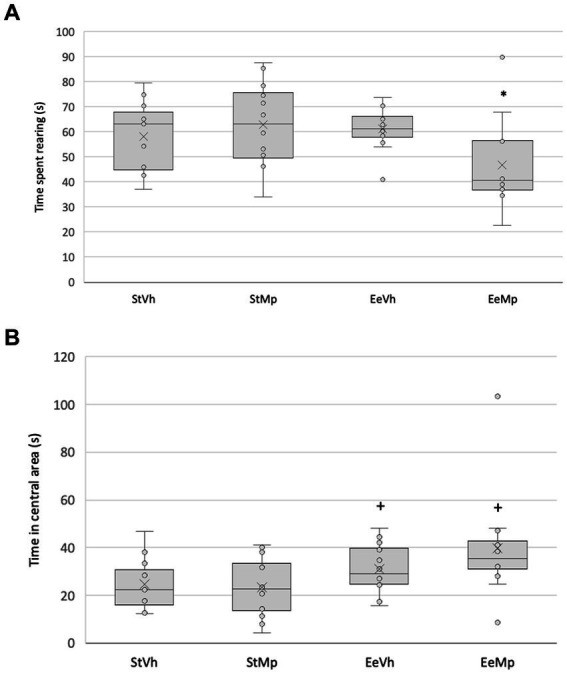
Time spent in explorative rearing behavior **(A)** and exploring the central area **(B)** of the open field for all groups. Median represented by the inner line in the box and Average represented by the inner “x”. +: different from the control environment housing condition with the same treatment.

The Kruskall-Wallis test found significant differences in rearing in the open field (H[3] = 9.625, *p* = 0.022, see Figure 3A). *Post hoc* comparison of group means using the Dunn’s method revealed that, in general, animals receiving methylphenidate and housed under the enriched condition explored less than those housed in the standard environment condition.

The Kruskal-Wallis test also found significant differences in exploration time in the central area (H[3] = 8.786, *p* = 0.032). *Post hoc* comparison of group means using the Dunn’s method revealed that, in general, animals housed in the enriched environment explored the central area more than those in the standard environment (see Figure 3B).

### Behavioral regulation

**Figure 4:**
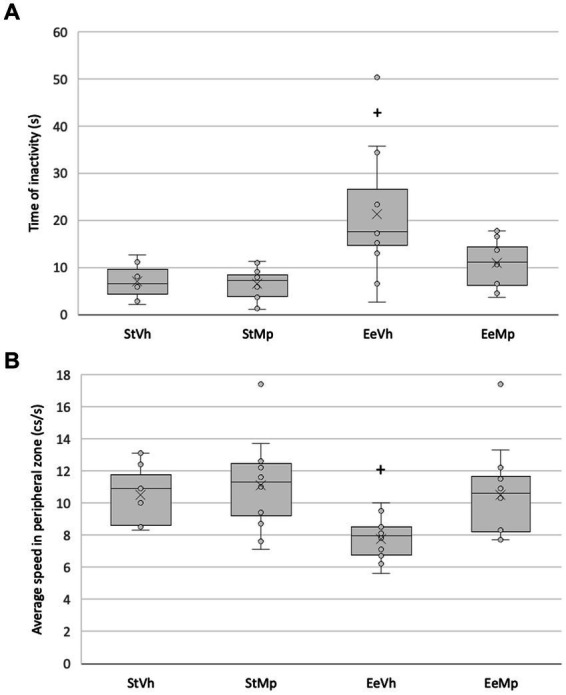
**(A)** total time of inactivity for all the subjects in the elevated plus maze and **(B)** average speed during the exploration of the periphery of the elevated plus maze. Median represented by the inner line in the box and Average represented by the inner “x”. +: different from the control environment housing condition with the same treatment.

The Kruskal-Wallis test showed significant differences for the inactivity time of the animals in the elevated plus maze (H[3] = 16.451, *p* < 0.001). *Post hoc* comparison of group means using the Dunn’s method revealed that in the enriched environment condition, animals that received methylphenidate were less inactive than those that received the vehicle (see Figure 4A).

Regarding the exploration speed of the periphery in the open field, the Kruskal-Wallis test found significant differences (H[3] = 13.714, *p* = 0.039). *Post hoc* comparison of group means using the Dunn’s method showed that for animals that received the vehicle, those in the enriched environmental condition exhibited a decreased exploration speed compared to those in the standard condition (see Figure 4B).

### Social behavior

**Figure 5:**
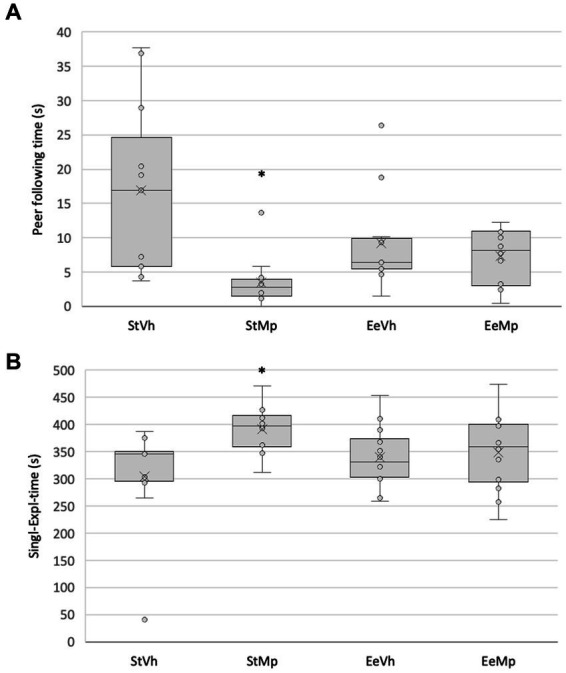
Total time spent **(A)** following another individual and **(B)** single exploration in the social interaction situation. Median represented by the inner line in the box and Average represented by the inner “x”. *: different from the vehicle in the same environment housing condition.

In the social interaction test, the Kruskal-Wallis test revealed significant differences (H[3] = 16.755, *p* < 0.001) in the time spent following another individual. The *post hoc* comparison of means using the Dunn’s method showed that animals housed in the standard environment condition and receiving methylphenidate had a lower amount of time spent chasing their companions (see Figure 5A).

The Kruskal-Wallis test found significant differences (H[3] = 9.041, *p* = 0.029) in the amount of time spent in single exploration. The *post hoc* comparison of group means using the Dunn’s method revealed that animals that received methylphenidate and were housed in the standard environment had longer durations of single exploration. This effect was not observed in animals that received methylphenidate and were housed in the enriched environment (see Figure 5B).

### Memory

No significant differences were found between the groups in the exploration time of the novel object in the object recognition test.

## Discussion

This study aimed to determine whether environmental enrichment can reverse or mitigate some of the effects of MPH on anxiety, locomotion, social interaction, and memory in Wistar rats. For this purpose, animals housed under standard or enriched conditions received chronic low-dose treatment of MPH or vehicle. The objective of our study was to investigate the long-term impact of chronic use of methylphenidate during early adolescence. To avoid interference from both the effect of methylphenidate and its withdrawal on the behaviors studied, it was decided to wait 7 days after the last administration, a period after which it can be ensured that there will be no interference from these two processes (Askenasy et al., 2007). Similarly, we sought to have a period of time not very different between the last administration of methylphenidate and the behavioral tests performed. For this reason, the behavioral testing was carried out with a little time of rest between them. In order to optimize the behavioral protocol used, the habituation session to the object recognition test was carried out in an open field arena so this session was used for both purposes, habituation to the place where the object recognition test was to be done and open field test.

There is no consensus in the literature regarding the potential interference of the order between different tests (Blokland et al., 2012), especially when working in mice (McIlwain et al., 2001; Võikar et al., 2004), nor on the best time that should be left between each of them. Blokland et al. (2012) reports that there may be an interference effect of the application of tests such as the Zero-Maze and the open field on performance in the forced swim test. Their results are understandable because, since the forced swim test is a test focused on the evaluation of depression, its performance can be interfered with by a stress effect after the application of other tests. In our case, neither the object recognition test nor the social interaction test have been reported as forms of measuring depression, nor as possible tests for measuring emotional factors, so an interaction effect between them with the elevated plus maze and the open field was not expected.

On the other hand, some authors propose that a period of 3 days between the completion of one test and the start of another is adequate (Sosa et al., 2019), when dealing with tests that involve large motivational loads for animals. In our case, none of the tests used causes emotional effects or induces emotional conditioning, so interaction between them is not plausible.

In particular, and due to the high sensitivity of the elevated plus maze for the measurement of anxiety (Hogg, 1996; Morato and Brandão, 1996), it is always chosen to perform it first and before any other. Similarly, and perhaps based on the initial work of Kostowski et al. (1989), it is common practice in many laboratories to sequentially evaluate behavioral tests that do not involve the use of painful or emotional learning stimuli (Kostowski et al., 1989), a situation that also reduces stress due to manipulation and ensures that there are not too many variations in the physiological conditions under which the behavioral evaluation is performed (Corredor et al., 2022).

Finally, it is worth mentioning that since control groups were used for each of the factors (environment and pharmacological treatment) and since all animals received exactly the same treatment and had exactly the same experimental times, any interference that any of these behavioral tests could have had on the subsequent ones would have also been present in the control groups, and therefore it can be ensured that this hypothetical effect would be present in all conditions.

To assess anxiety, we analyzed the time spent in the open arms of the EPM. Some studies have reported a moderate anxiolytic-like effect of environmental enrichment (Corredor et al., 2022). However, paradoxically, our results showed that the increased exploration of the open arms observed in animals housed under standard conditions and treated with MPH was reversed by the enriched environment. This suggests a paradoxical anxiogenic effect of the enriched environment. However, it is possible that the increased exploration of the open arms in animals treated with MPH does not indicate an anxiolytic effect but rather a lack of behavioral regulation, indicative of impulsive-like behavior. If this is the case, the paradoxical effect of the enriched environment could be better understood as a reversal of the impulsive behavior induced by MPH. The lack of effect of MPH on the time spent exploring the central area of the open field supports the notion that it does not reflect a genuine decrease in anxiety. While time spent in open arms is often interpreted as a reduction in anxiety, it’s important to continue exploring other potential explanations that could account for this phenomenon. For instance, within the context of our study, when linking environmental enrichment with the consumption of a psychostimulant drug wherein the environment mitigates the drug’s effect, it’s plausible that the mitigated impact pertains to the stimulant effect on animal locomotion. This mitigation could lead to an increase in impulsive movements, which are typically suppressed by anxiety. We also consider the possibility that the drug, by inducing impulsivity, diminishes the animal’s risk assessment facilitated by anxiety. Consequently, this could explain why the animal spends more time in the open arms of the elevated plus maze. As highlighted in previous studies, it is crucial to consider that the impact of MPH on anxiety is influenced, to some extent, by the specific anxiety-inducing stimuli (Crawford et al., 2013). Therefore, conducting a more comprehensive investigation into the mechanisms underlying the heightened stimulation of the open arms induced by MPH would be valuable.

Moreover, it could be suggested that the increase in time spent in the open arms is associated with locomotion and reactivity to novelty, as proposed by certain authors (Dow-Edwards et al., 2008). However, this does not appear to be the case in our study. In fact, our results demonstrated no difference in total distance covered in the elevated plus maze, nor in the average exploration speed in the open field, suggesting the absence of any MPH-induced effects on locomotion. Nevertheless, further exploration of impulsive behavior in the elevated plus maze is needed.

Rearing behavior, which involves the animal raising its front legs and relying solely on its hind legs, is commonly used as a measure of exploration (Sturman et al., 2018). Our results indicate that housing animals treated with MPH in an enriched environment reduces the tendency to engage in rearing behavior. This unexpected effect could suggest that MPH-treated animals are more susceptible to the effects of environmental enrichment. However, it is crucial to delve into how the enriched environment might be mitigating the drug’s impact on the animal’s exploratory behaviors. In the event that methylphenidate consumption enhances exploratory behavior such as rearing, this effect could also be attributed to impulsive motion, and conceivably, the enriched environment could be counteracting the impulsivity induced by the drug. Nonetheless, there is a need to continue investigating various motor and emotional variables that could drive an animal to explore an environment, in order to better comprehend the circumstances under which this could be beneficial or not for the animal. In our study, we contend that irrespective of the reasons behind the animal’s engaging in the behavior, the environment is ameliorating the drug’s effects, thus bolstering the notion that the environment can function as a protective factor against substance consumption effects. Further research is necessary to explore exploratory behaviors like rearing and other variables that may be linked to these behavioral changes. Lastly, since this experiment used rats without any attentional disorder, it raises the question of how MPH would affect exploratory rearing in rats with attentional impairments.

Certain authors have demonstrated that MPH and atomoxetine can inhibit social gambling behavior by altering the noradrenergic system (Achterberg et al., 2015). While this study assesses behaviors other than gambling, its objective is to evaluate the impact of MPH on the fundamental social interaction of animals.

Social behavior was assessed using a test that enabled free interaction while also providing the option to maintain distance from others. By observing specific social interactions, behaviors such as physical contact between animals, interest in proximity to others, the intention to initiate social interaction, and preference for individual exploration can be measured. One particular behavior, known as “following behavior,” involves rodents initiating social interaction by approaching and sniffing the anogenital area of another animal (Meaney et al., 1981).

Our findings demonstrated that chronic MPH significantly inhibits the initiation of social behavior. Additionally, we observed that MPH administration increased the time spent in solitary exploration. However, when animals treated with MPH were housed in an enriched environment, the duration of social behavior (specifically, “following behavior”) was comparable to that of animals receiving the vehicle. The same is true for single exploration.

From these results, it is evident that the enriched environment has the ability to reverse the decreased interest in initiating social contacts. It is noteworthy that when comparing the time spent following other peers between animals housed under the two different conditions, the enriched condition resulted in less variability compared to the standard housing condition. In future studies, it would be intriguing to explore neurobiological changes that can shed light on the mechanisms through which the enriched environment manages to counteract the drug’s impact on the social interaction of animals. Given that this study solely observed behavior, it allows us to speculate that once again, the enriched environment serves as a protective factor against the impact of addictive drug consumption on the social interaction of the animals, through a modulating effect on anxiety. In this context, if methylphenidate is consumed within an enriched environment, it could potentially reverse the decrease in social interaction induced by the drug. However, the implications of this would vary depending on the circumstances, as it hinges on whether harnessing the drug’s effect is required or advantageous for the animal within a specific context. It is crucial to highlight that social behavior serves as an adaptive function in mammals, and it is directly linked to survival and reproduction in adulthood (Achterberg et al., 2015). Furthermore, social experiences during adolescence play a fundamental role in the social, cognitive, and emotional development of rats (Hodges et al., 2018).

Additionally, it is worth noting that in humans, the inhibition of social behaviors may be one of the factors contributing to the efficacy of MPH as a treatment for ADHD, as it reduces attention to environmental stimuli (Vanderschuren et al., 1997). Therefore, the observed effect of the enriched environment on social interaction needs to be carefully considered, as it could have unintended consequences or be seen as an undesired effect.

In relation to locomotion, our findings revealed that environmental enrichment has a regulatory effect on behavior. Specifically, rats housed in the enriched condition exhibited increased time of inactivity (distinct from freezing) in the elevated plus maze. This inactivity could be interpreted as a strategy to regulate impulsive behavior, allowing the animals to better consolidate acquired information. This interpretation aligns with the observed effect of MPH on open arm exploration. If this is the case, our results suggest that the enriched environment was unable to counteract the impulsive behavior induced by MPH. In fact, rats receiving MPH and housed in the enriched condition did not exhibit an enhanced duration of inactivity. It is also plausible that the mechanism through which inactivity regulates impulsivity is related to reduced levels of anxiety (Holubová-Kroupová and Šlamberová, 2021).

The pseudo-anxiolytic-like effect observed with MPH may potentially indicate a compensatory mechanism in response to chronic drug consumption (Crawford et al., 2013). In this scenario, it could imply a general increase in motor activity without a specific exploratory aim.

In relation to memory, although there were no significant differences in the interaction between the pharmacological treatment and housing condition, there are noteworthy findings to highlight. Firstly, environmental enrichment as a standalone factor appeared to enhance memory by increasing the exploration time of the novel object. This finding aligns with previous studies suggesting that an enriched environment can improve memory by facilitating cognitive changes (Leger et al., 2015).

Secondly, although it is striking that unlike most other studies, MPH did not induce any memory changes in our experiment. In other studies, even at low doses, MPH has been shown to enhance memory (Carmack et al., 2014). Therefore, it is essential to consider additional environmental variables that may interfere with the results and influence the effects of MPH on memory.

We propose the possibility that the lack of MPH effect on memory in our study could be attributed to the specific protocol used for the object recognition task. Unlike most other studies, our protocol focuses on measuring declarative memory rather than spatial memory, as it involves changing the object itself rather than its location. It is important to mention that this protocol has been standardized and utilized by the Laboratory of Neuroscience and Behavior at the University of Los Andes (documentation available at the university’s library repository).

Another aspect to take into consideration is the interval that elapsed between the completion of drug administration and the commencement of the experiments. Variables such as the number of days of drug administration and the withdrawal period need to be accounted for in future research to determine the implementation of environmental enrichment protocols and behavioral observation. Further studies are necessary to attain more distinct conclusions regarding the impact of methylphenidate on memory.

## Data availability statement

The raw data supporting the conclusions of this article will be made available by the authors, without undue reservation.

## Ethics statement

The animal study was approved by IACUC – Universidad de los Andes. The study was conducted in accordance with the local legislation and institutional requirements.

## Author contributions

All authors listed have made a substantial, direct, and intellectual contribution to the work and approved it for publication.
