# Erratum: Role of environmental enrichment on social interaction, anxiety, locomotion, and memory in Wistar rats under chronic methylphenidate intake

**DOI:** 10.3389/fnbeh.2024.1431914

**Published:** 2024-05-20

**Authors:** Frontiers Production Office

**Affiliations:** Frontiers Media SA, Lausanne, Switzerland

**Keywords:** methylphenidate, psychostimulants, social interaction, anxiety, enriched environment, ADHD, rat

Due to a production error, there was an error in the authors' affiliation. Instead of “Laboratory of Neuroscience and Behavior, Department of Psychology, Universidad de los Andes, Bogotá, CO, United States”, it should be “Laboratory of Neuroscience and Behavior, Department of Psychology, Universidad de los Andes, Bogotá, Colombia”.

The publisher apologizes for this mistake. The original article has been updated.

